# Supplementary figures and images for: Development of a method of passaging and freezing human iPS cell-derived hepatocytes to improve their functions
Source: PLoS One. 2023 May 18;18(5):e0285783. doi: 10.1371/journal.pone.0285783 (PMC10194907; doi:10.1371/journal.pone.0285783)

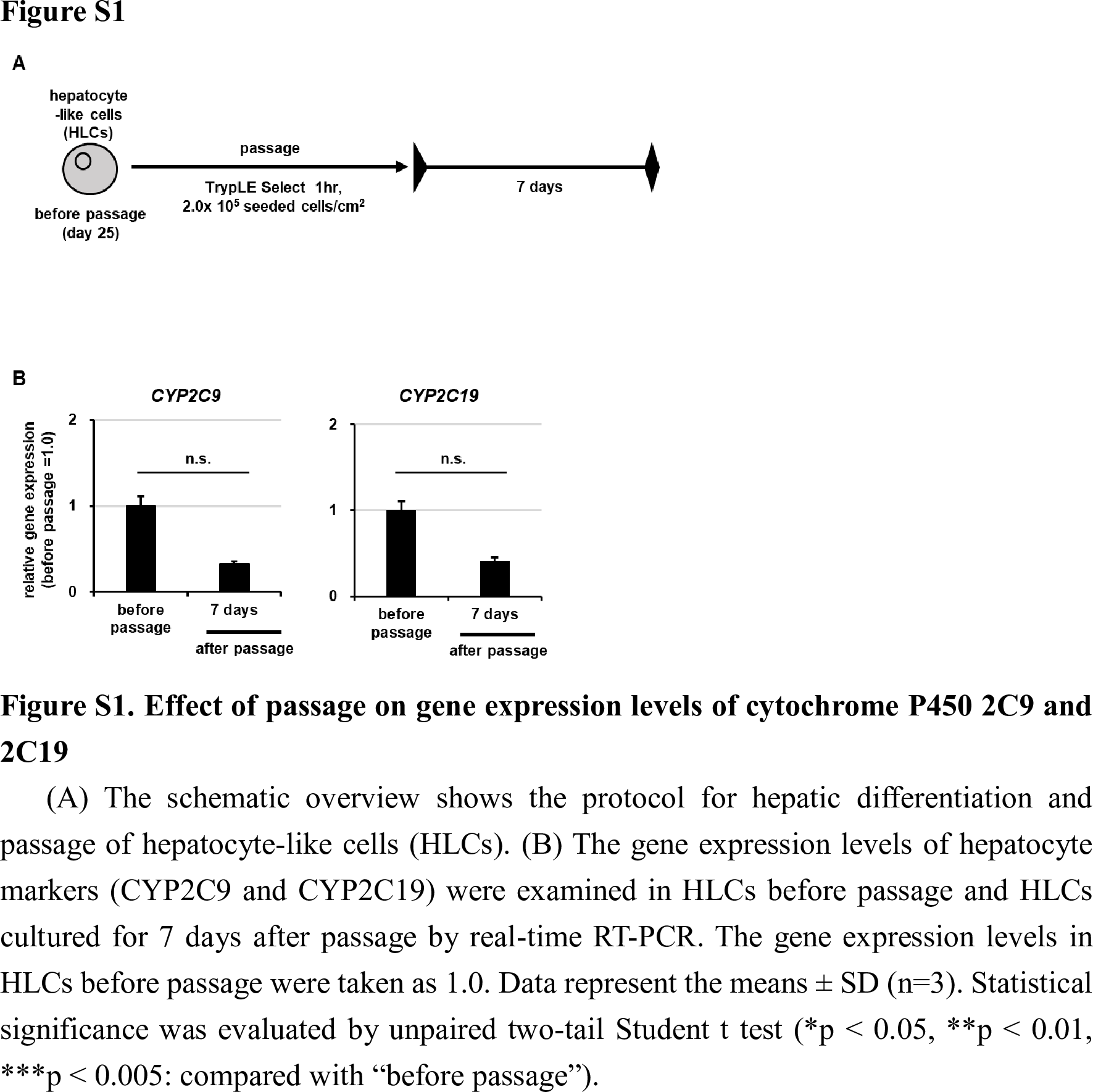

Supplement: S1 Fig — (TIF) [file pone.0285783.s001.tif]

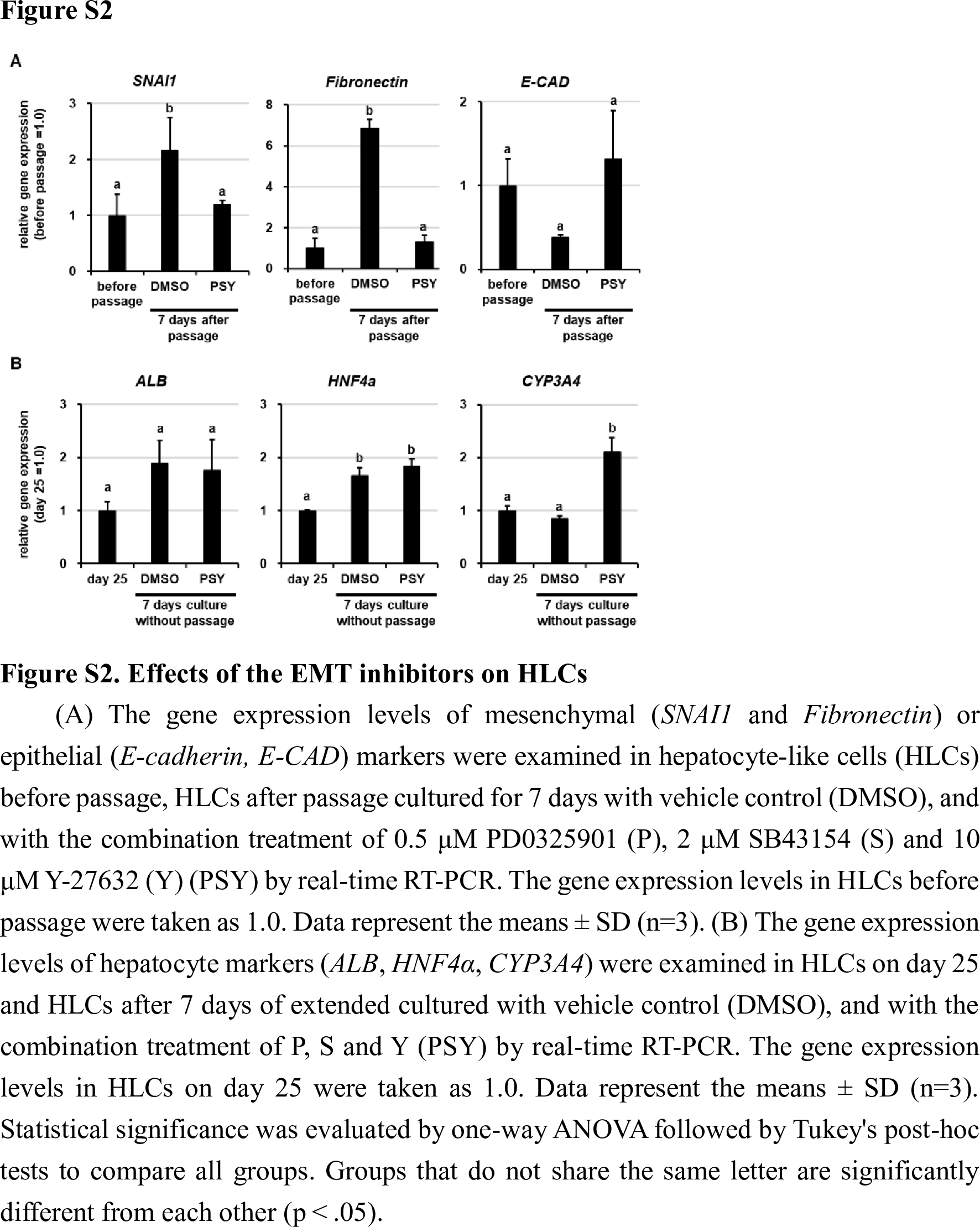

Supplement: S2 Fig — (TIF) [file pone.0285783.s002.tif]

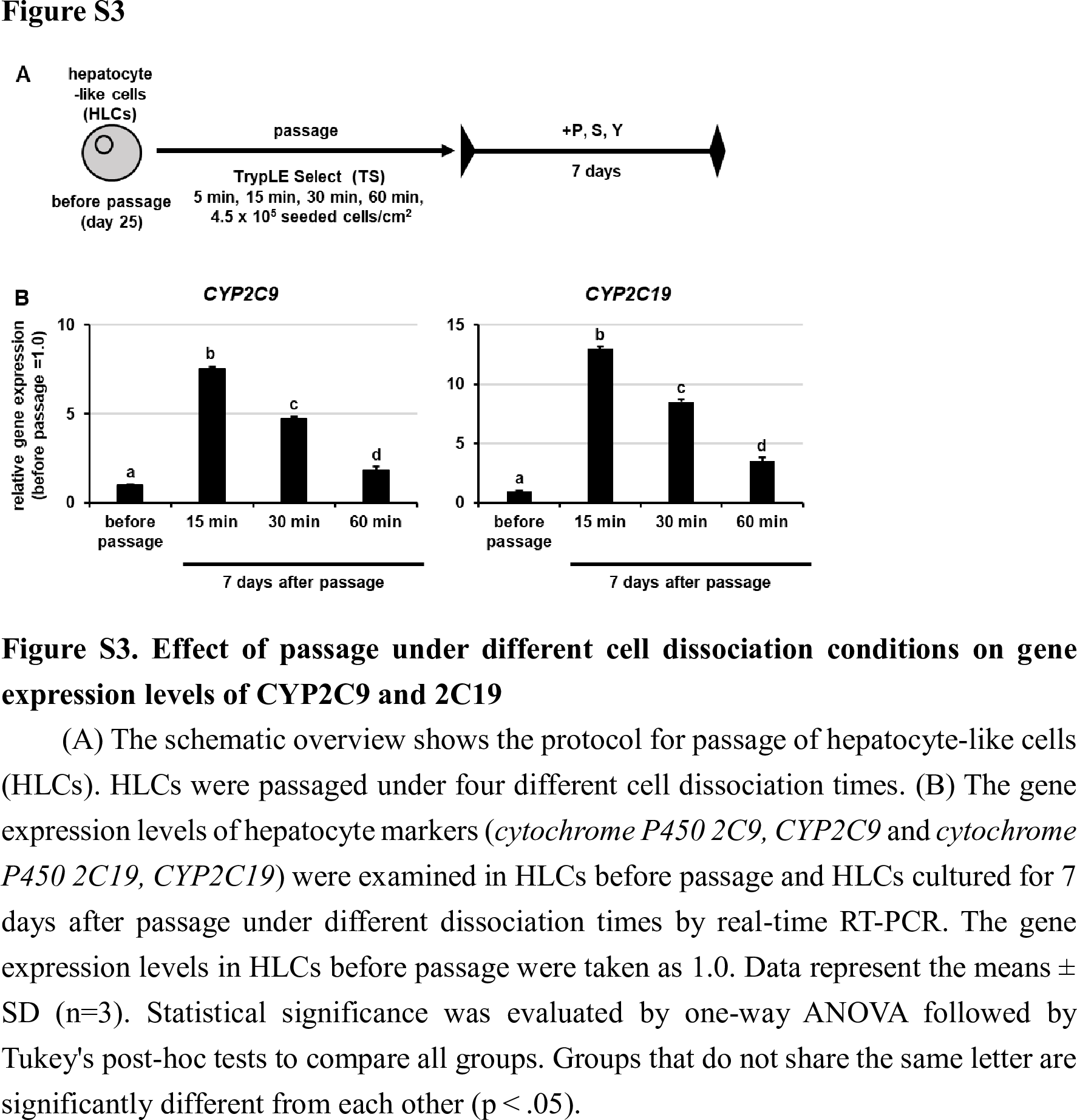

Supplement: S3 Fig — (TIF) [file pone.0285783.s003.tif]

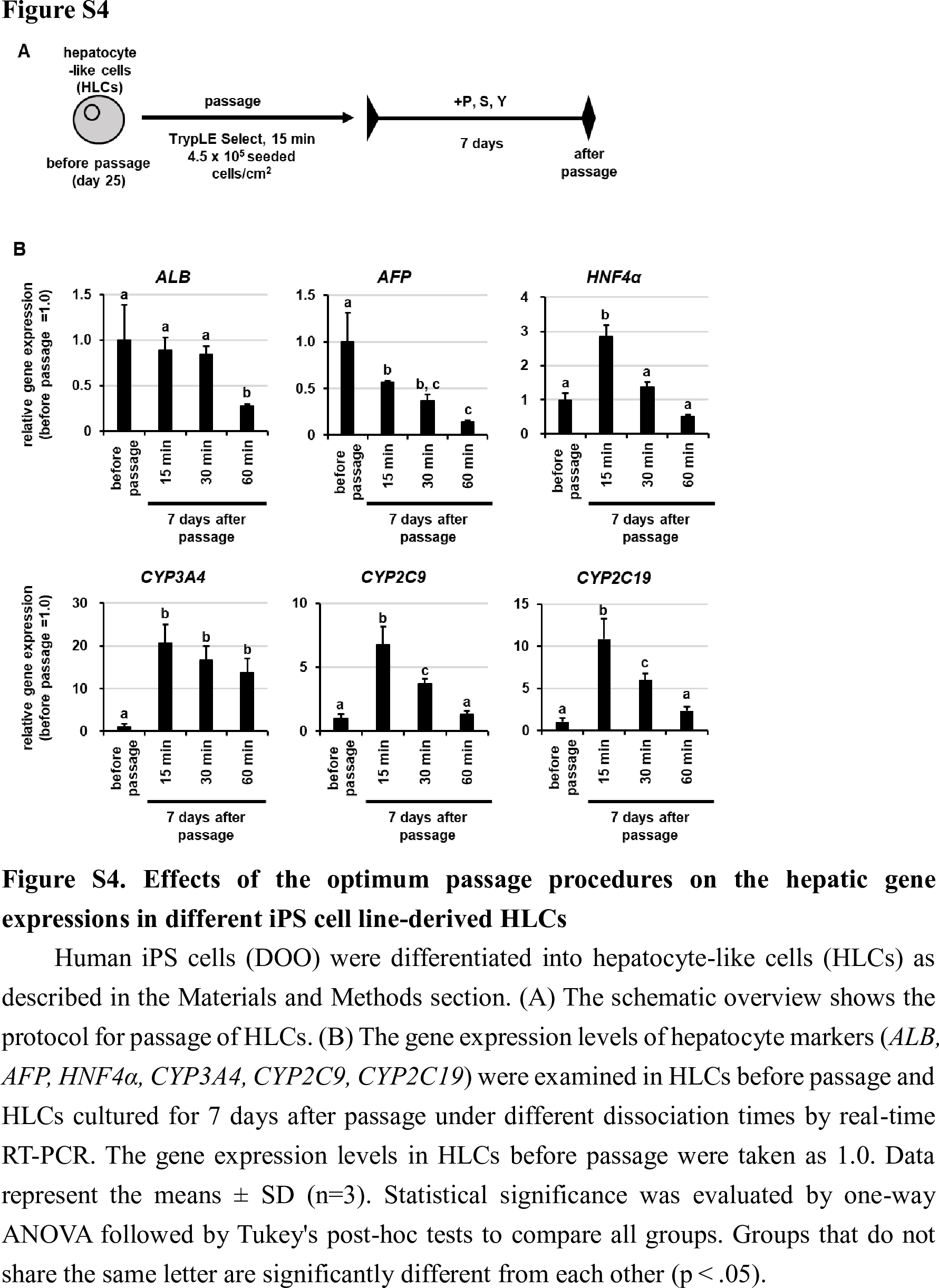

Supplement: S4 Fig — (TIF) [file pone.0285783.s004.tif]

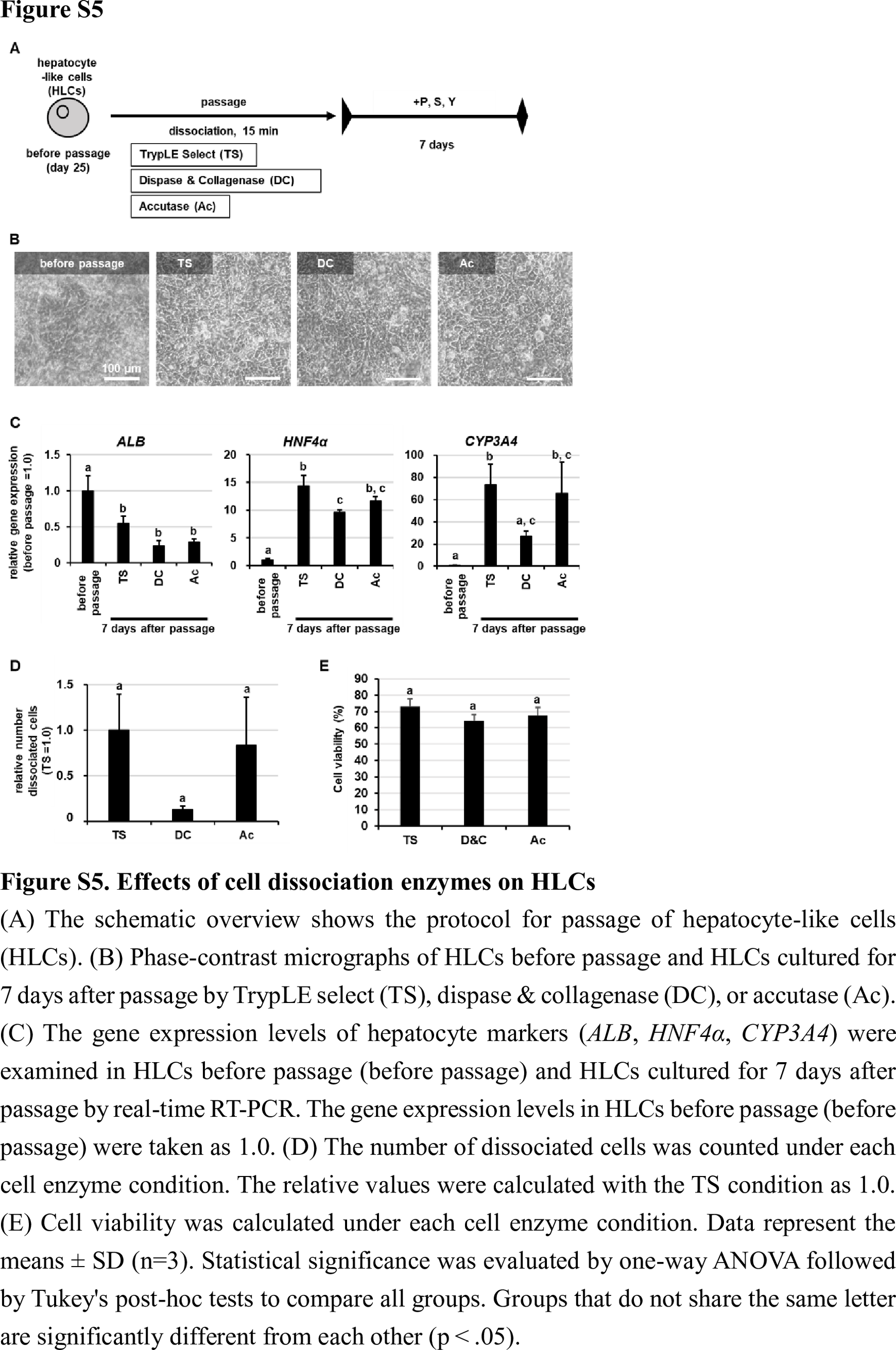

Supplement: S5 Fig — (TIF) [file pone.0285783.s005.tif]

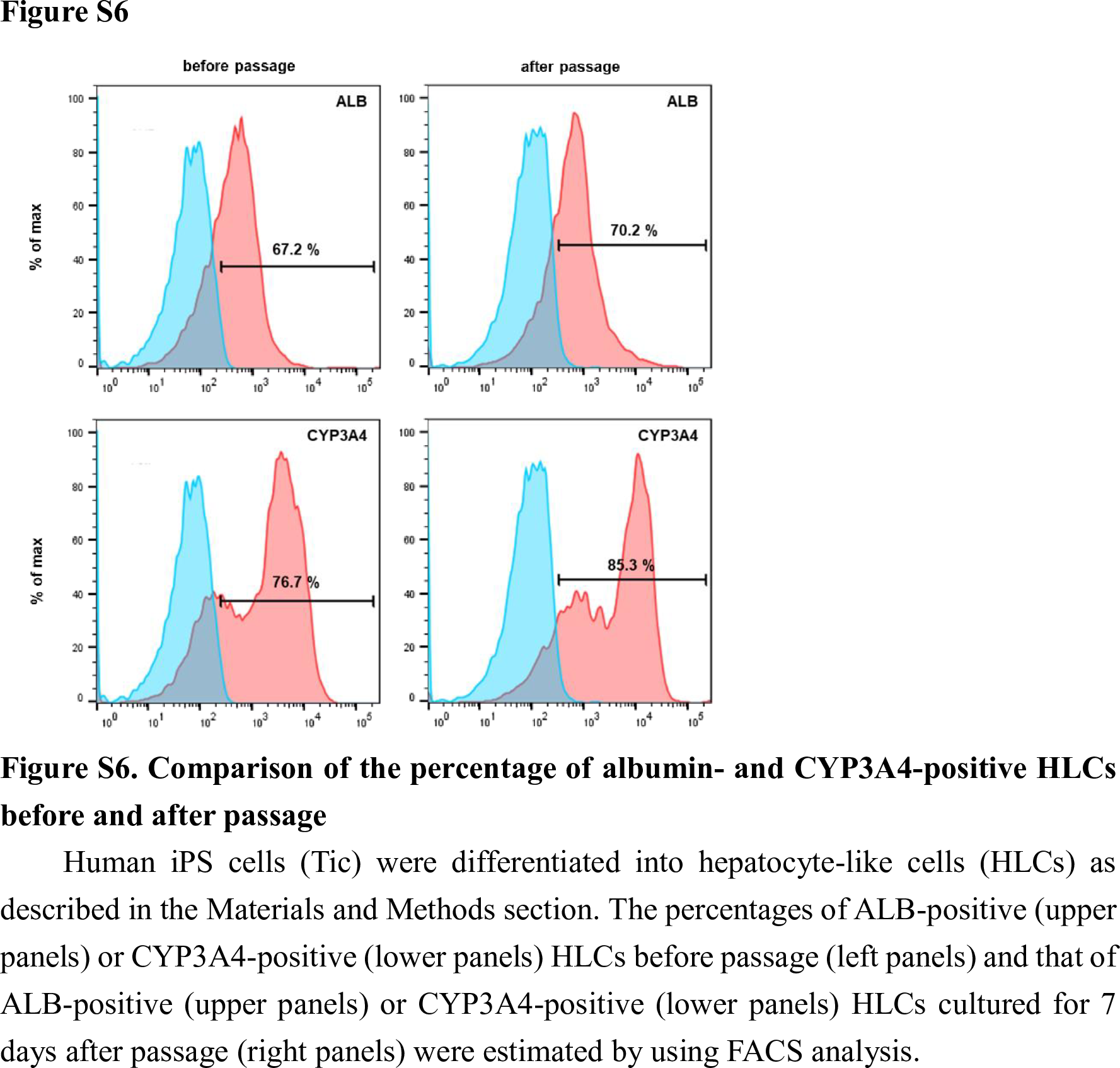

Supplement: S6 Fig — (TIF) [file pone.0285783.s006.tif]

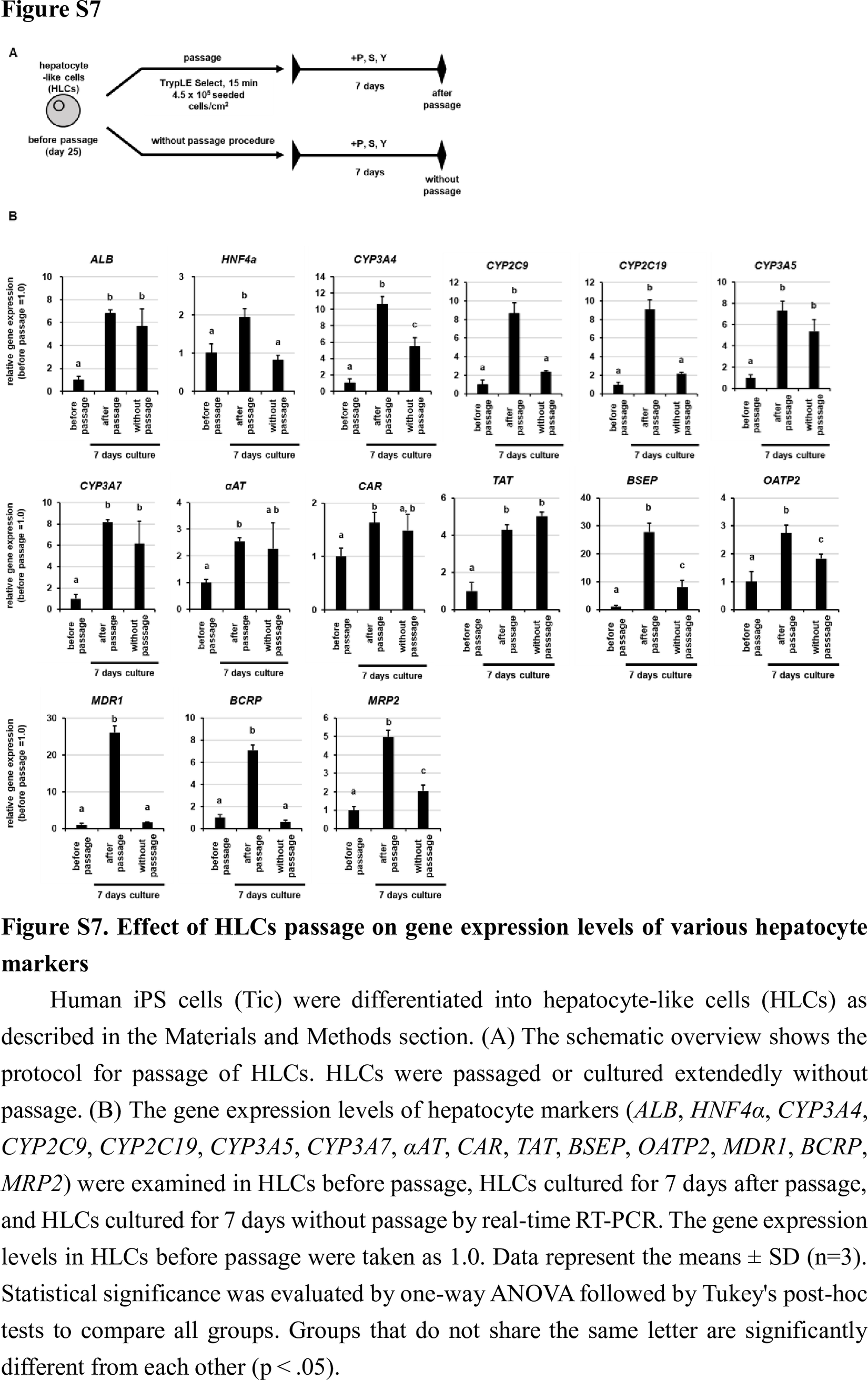

Supplement: S7 Fig — (TIF) [file pone.0285783.s007.tif]

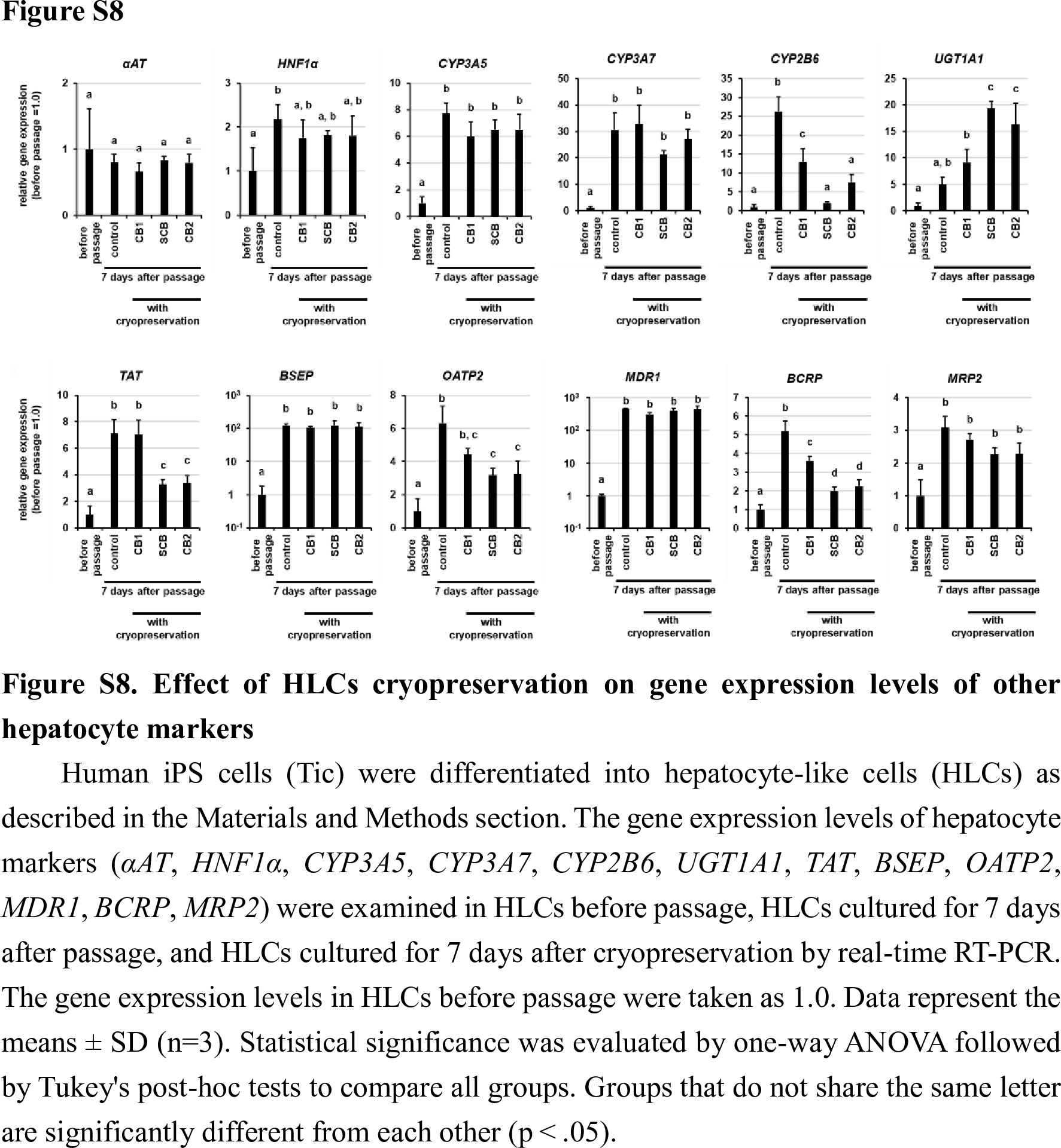

Supplement: S8 Fig — (TIF) [file pone.0285783.s008.tif]

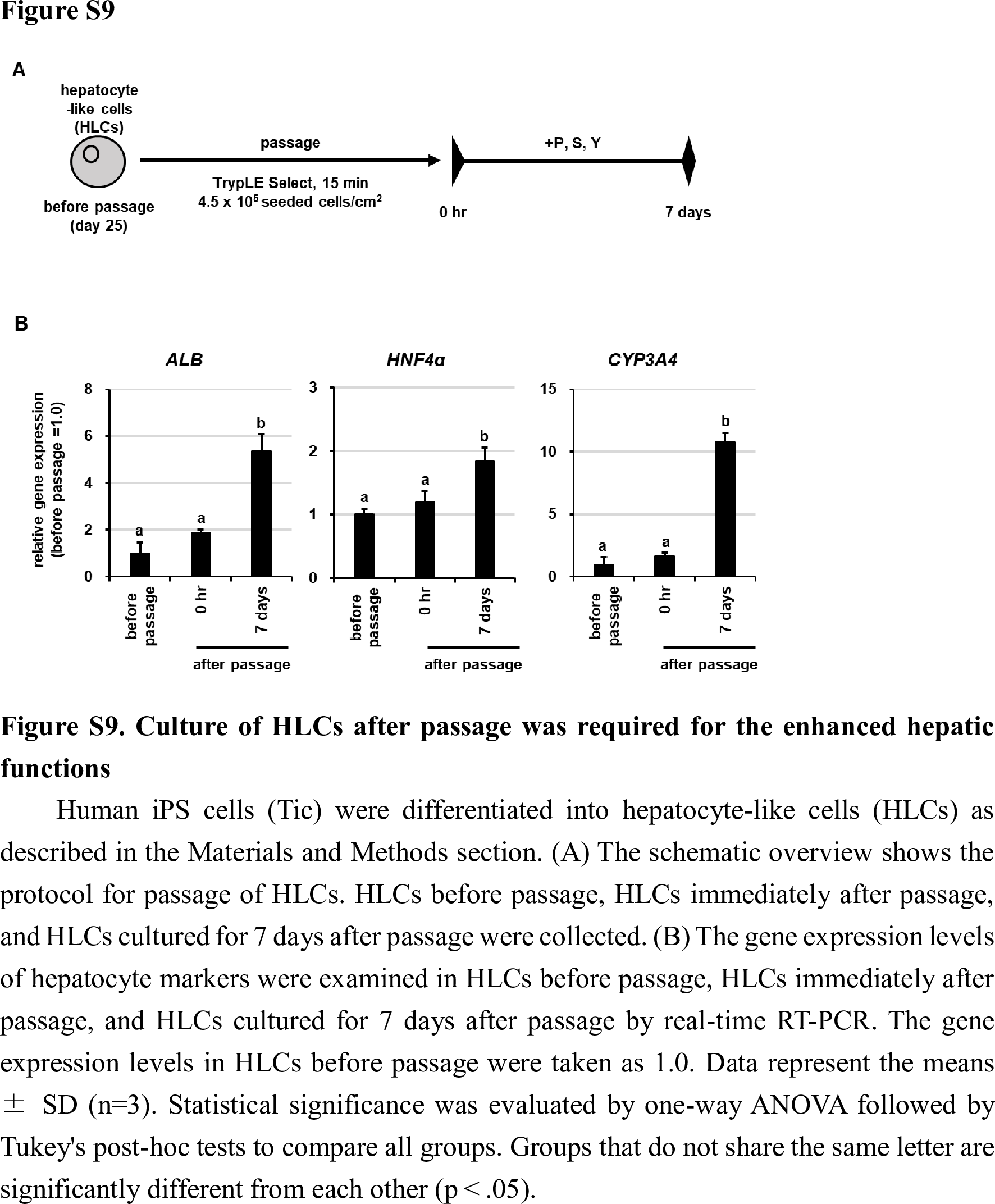

Supplement: S9 Fig — (TIF) [file pone.0285783.s009.tif]

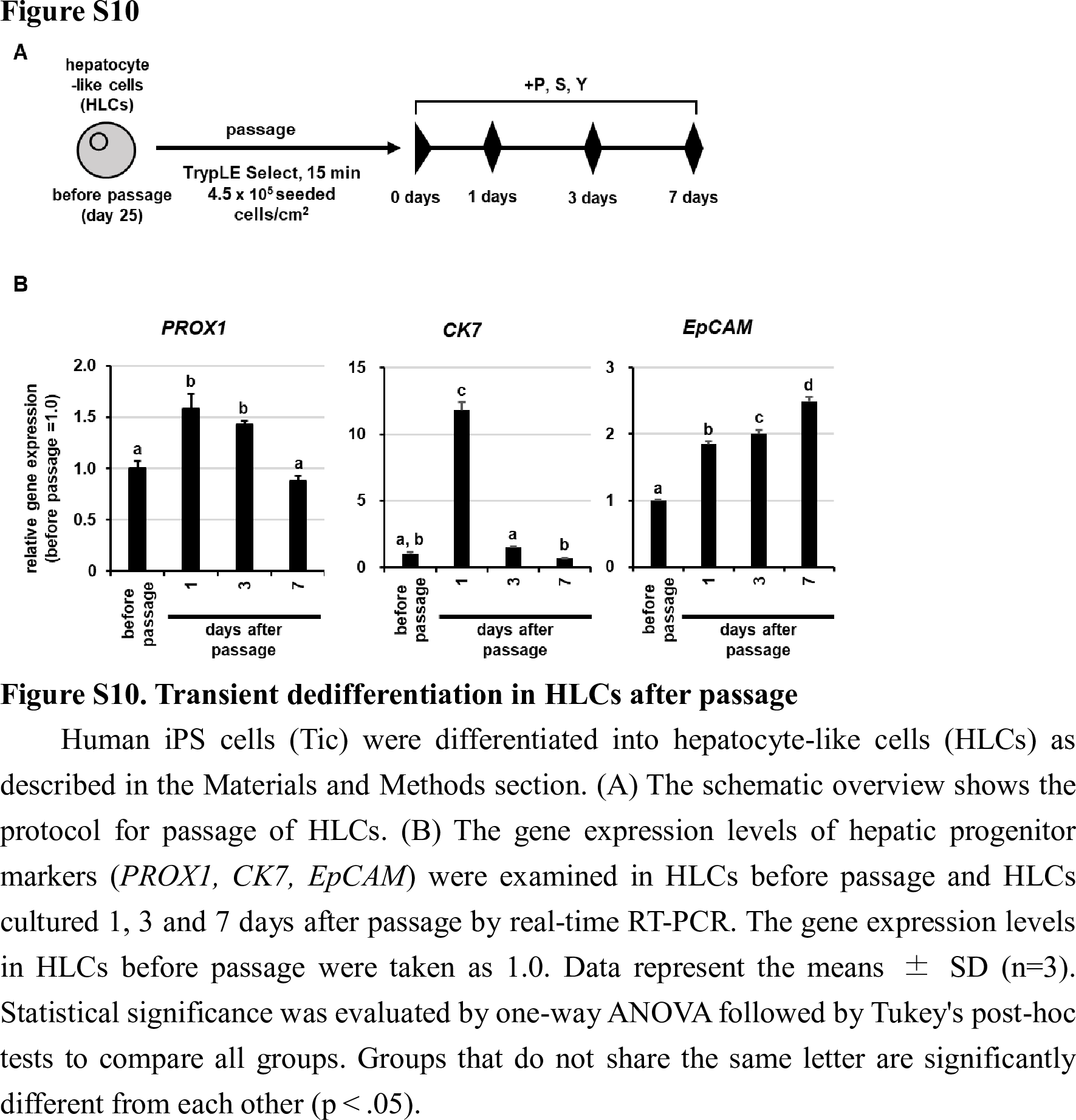

Supplement: S10 Fig — (TIF) [file pone.0285783.s010.tif]
