# Supplementary material for: Development of a method of passaging and freezing human iPS cell-derived hepatocytes to improve their functions
Source: PLoS One. 2023 May 18;18(5):e0285783. doi: 10.1371/journal.pone.0285783 (PMC10194907; doi:10.1371/journal.pone.0285783)
Supplement: Attachment — Submitted filename: Response to Reviewers.docx [file pone.0285783.s011.docx]

**Dear Editor of PLoS ONE**

Thank you for your effort in reviewing our manuscript entitled “Development of a method of passaging and freezing human iPS cell-derived hepatocytes to improve their functions.” by J. Inui et al. We thoroughly revised the manuscript in keeping with editor’s and reviewers’ suggestions. The sentences we revised to the manuscript are shown in red. Point-by-point responses about editor’s and reviewers’ suggestions are given below.

**Changes required for acceptance:**

**1. Functional assays such as albumin ELISA and urea assay should be performed.**

*Response:*

We compared albumin secretion, urea secretion, and CYP3A4 activity in HLCs after passage or cryopreservation in CB1 with those in HLCs before passage. The results showed that HLCs had these functions after passage or cryopreservation comparable to or better than those in HLCs before passage.

Please see lines 216-221 and Fig.7.

**2. Quantification of immunostaining results should be presented (e.g. Fig. 4C, D, E).**

*Response:*

We found it difficult to quantify the immunofluorescent staining images. Therefore, we instead performed FACS analysis about the expression of the most important hepatocyte markers, ALB and CYP3A4. The results showed that the percentage of ALB- or CYP3A4-positive cells increased in the HLCs after passage compared to before passage. Please see lines 170–174 and Fig.S6.

In the results in Fig. 4D and E, the analyses were performed to confirm that HLCs after passage still had the capacity for glycogen storage and LDL uptake, and did not indicate that these functions were higher than before passage.

**Recommended changes:**

**1. The quality of figures should be improved.**

*Response:*

We corrected the data with increased resolution.

**2. Alternative cell dissociation methods should be explored.**

*Response:*

We showed the results using other dissociation enzymes [dispase & collagenase (DC), TrypLE select (TS) and accutase (Ac)] in Fig. S5, and decided to use TrypLE select, which showed a more favorable trend, although not significantly different. We tried to dissociate HLCs with 5 minutes treatment of TrypLE select, but the cells were not recovered efficiently enough to perform the experiments, as shown in Fig. 3B.

**3. Other recommended changes as suggested by the reviewers.**

*Response:*

We thoroughly revised the manuscript and figures in keeping with each reviewer's suggestions. Point-by-point responses about reviewers’ suggestions are given below.

**Reviewer #1:**

1. **Fig4C lacks quantification and does not compare the ratio of ALB HNF4A CYP3A4 AAT before and after passaging. neither does Fig4D and E.**

*Response:*

　As the reviewer suggested, we performed FACS analysis to determine the percentage of ALB- or CYP3A4-positive cells before and after passage. The results showed that the percentage of ALB- or CYP3A4-positive cells increased in the HLCs after passage compared to before passage. Please see lines 170–174 and Fig.S6.

In the results in Fig. 4D and E, the analyses were performed to confirm that HLCs after passage still had the capacity for glycogen storage and LDL uptake, and did not indicate that these functions were higher than those before passage.

2. **Fig4B suggests using ES as a negative control and Fresh PHH as a positive control.**

*Response:*

Since HLCs are derived from human iPS cells, we measured CYP3A4 activity in human iPS cells as a negative control and found no activity (data not shown). Most pharmaceutical companies perform drug toxicity studies with PHHs cultured for 48 to 72 hours. Therefore, in this study, we used PHHs cultured for 48 hours as a control to demonstrate the usefulness of HLCs after passage for drug toxicity studies.

3. **The full text should be quantified using the ALB Secretion ELISA assay.**

*Response:*

　As the reviewer suggested, we compared the levels of albumin secretion in HLCs after passage or cryopreservation with those in HLCs before passage. The results showed that the levels of albumin secretion increased significantly in HLCs after passage, and this trend was maintained even after cryopreservation. Please see lines 216-221 and Fig.7.

4. **Fig6, Comparison of cell activity after recovery with different cryopreservation solutions. In addition to qPCR, more functional quantitative data should be used for comparison.**

*Response:*

　As the reviewer suggested, we compared albumin secretion, urea secretion, and CYP3A4 activity in HLCs after passage or cryopreservation in CB1 with those in HLCs before passage. The results showed that HLCs after cryopreservation had these functions comparable to or better than the HLCs before passage, although urea secretion and CYP3A4 activity in the HLCs after cryopreservation were lower than those in the HLCs after passage. Please see lines 216-221 and Fig.7.

**Reviewer #2:**

1. **The quality of the figures should be deeply improved. The schemes used are so simplistic and could be also improved and make them more appealing.**

*Response:*

We corrected the data with increased resolution.

2. **The authors just analyzed the use of TriplE for passaging the cells although many authors have used other systems such as collagenase, dispase or cell dissociation buffer for cell passaging. The authors should explore the use of other methods that just require 5-10 minutes.**

*Response:*

We showed the results using other dissociation enzymes [dispase & collagenase (DC), TrypLE select (TS) and accutase (Ac)] in Fig. S5, and decided to use TrypLE select, which showed a more favorable trend, although not significantly different. We tried to dissociate HLCs with 5 minutes treatment of TrypLE select, but the cells were not recovered efficiently enough to perform the experiments, as shown in Fig. 3B.

3. **A more detailed transcriptomics analysis (at least 10 genes) would improve the quality of the manuscript.**

*Response:*

　As the reviewer suggested, we additionally examined the gene expression levels of more than 10 hepatocyte markers. Most genes were highly expressed in HLCs after passage or cryopreservation compared to those in HLCs before passage. Please see lines 185-187, 213-215, and Fig.S7 & S8.

4. **Functionality of the cells in terms of albumin production and secretion and ureogenesis capability should be also included since these are specific hepatic functions that are normally analysed in HLC.**

*Response:*

　As the reviewer suggested, we compared albumin secretion and urea secretion in HLCs after passage or cryopreservation in CB1 with those in HLCs before passage. The results showed that HLCs had these functions after passage or cryopreservation comparable to or better than those in HLCs before passage, although urea secretion in the HLCs after cryopreservation was lower than that in the HLCs after passage. Please see lines 216-221 and Fig.7.
